# The Influence of Fertilization and Plant Density on the Dry Matter Yield and Quality of Black Mustard [*Brassica nigra* (L.) Koch]: An Alternative Forage Crop

**DOI:** 10.3390/plants11202683

**Published:** 2022-10-12

**Authors:** Stella Karydogianni, Ioannis Roussis, Antonios Mavroeidis, Ioanna Kakabouki, Evangelia Tigka, Dimitrios Beslemes, Panteleimon Stavropoulos, Nikolaos Katsenios, Eleni Tsiplakou, Dimitrios Bilalis

**Affiliations:** 1 Laboratory of Agronomy, Department of Crop Science, Agricultural University of Athens, 11855 Athens, Greece; 2 Institute of Industrial and Forage Crops, Hellenic Agricultural Organization Demeter, 41335 Larissa, Greece; 3 Department of Soil Science of Athens, Institute of Soil and Water Resources, Hellenic Agricultural Organization—Demeter, 14123 Attica, Greece; 4 Laboratory of Nutritional Physiology and Feeding, Department of Animal Science, School of Animal Biosciences, Agricultural University of Athens, 11855 Athens, Greece

**Keywords:** compost, crude protein content, nitrification inhibitor, nutritive value, seaweed compost, urease inhibitor, total carbohydrate content

## Abstract

Black mustard [*Brassica nigra* (L.) Koch] is mainly cultivated as a seed crop, and there is a lack of information on biomass quality and its potential for animal feeding. A 2-year field experiment was set up in a split-plot design with 2 main plots (plant densities: 46 and 76 plants m^−2^), 4 sub-plots (fertilization levels: control, compost, urea with and without urease and nitrification inhibitors) and 3 replications for each treatment. The highest dry matter yield (17.55–18.34 tn ha^−1^) was observed in high-density plots fertilized with urea fertilizer coated with double (nitrification and urease) inhibitors. In terms of the qualitive parameters of total above-ground biomass, the highest crude protein (CP) content was achieved in plots with low density and urea with double inhibitors. Moreover, the highest neutral detergent fiber (NDF) and acid detergent fiber (ADF) contents of above-ground biomass were found under compost and urea with double inhibitors. The high ADF, NDF and relatively high CP content characterized that black mustard aerial biomass can meet the requirements of lactating animals, and therefore the production of black mustard biomass as a forage crop could be of great importance. As a conclusion, black mustard cultivated at plant densities higher than 46 plants m^−2^ and under inorganic fertilization, especially with urea coated with double inhibitors, could be successfully used as a novel forage crop in ruminants’ diets.

## 1. Introduction

As the global population is expected to reach 9.8 billion by 2050 and 11.2 billion by 2100, there will be an increase in food demand [1]. It is predicted that global meat consumption will increase by approximately 73% by 2050, while dairy production will increase by 58% [2]. In this scenario, global animal production will confront considerable challenges in meeting the rising demand for animal protein over the next few decades. Sustainable production, efficient use of natural resources, and improved animal welfare are all crucial elements to consider when seeking to meet this intense demand [3]. As a result, animal nutrition can play a significant role in meeting the aforementioned needs. Higher animal production efficiency and improved body composition are significantly associated with animal nutrition, and maintaining dietary nutrition during important stages of animal life can have an impact on livestock productivity [4].

Nutrition can be a severe constraint to livestock productivity in general, especially when feed resources are insufficient in both quality and quantity. In the past, a growth in animal population was not necessarily followed by an increase in feed resource availability, which could result in decreased animal performance and health [5,6]. Furthermore, the increased demand for livestock products puts a strain on the requirement for high-producing animals. Inadequate feed quality and quantity inhibits improved animal output [7]. As a result, the rising demand for feed protein needs research into alternate feed sources with high nutrient content that are safe and may be added into animal diets to improve animal health and production.

Mustards are members of the Cruciferae or Brassicaceae family. There are 150 species of annuals or biennial herbs in the genus *Brassica*, many of which are produced as oilseed crops, vegetables, or fodder. Black mustard [*Brassica nigra* (L.) Koch] is an herbaceous annual plant with an unknown native range; however, it is most likely endemic to the southern Mediterranean region [8]. A black mustard plant has a solid taproot, huge lower leaves, smaller higher leaves, and a stem that is delicately covered with soft hairs. Black mustard differs from commercial *Brassica* crops in that it does not generate a rosette of basal leaves. The seeds of black mustard are globular in shape, 1–1.6 mm in diameter, dark brown to practically black in color, minutely reticulate, and mucilaginous [9].

Due to the extreme pungency of its seeds and its value as a leaf vegetable, black mustard was one of the first domesticated crops and was widely cultivated in central and southern Europe, North Africa, and Asia. In addition to its culinary application, the therapeutic potential of black mustard was recognized early on, with mustard meal being used to treat skin ailments, arthritis, and rheumatism [8,10]. Black mustard cultivation has recently gained popularity, and it has been transferred to countries other than its native continents, such as Australia and America, as a source of edible oil and seeds [11]. This crop has been chosen for its ability to grow in a variety of agroecological conditions, including relatively low temperatures and disturbed soils, making it well suited for cultivation for both domestic and industrial purposes [12]. Furthermore, black mustard production is predicted to increase more significantly in the next years owing to the increased use of mustard seeds in the food and beverage, pharmaceutical, personal care, and cosmetic industries [13]. In addition to the high quality of its oil, its seed flour is high in protein and is even ideal for use in animal husbandry as an additive in animal feed, especially for poultry [14]. Finally, black mustard seed meal, the residual by-product of pressed black mustard seed, can also be utilized safely and economically in animal feeding as a relatively good source of energy and protein [15].

Nitrogen (N) is the most important nutrient for plant growth, development, and quality, as well as the most complex, due to the multiple forms and activities that might occur during its cycle [16]. It plays an important role in all plant metabolic activities, and its rate of absorption and partitioning is principally influenced by supply and demand during the plant’s life cycle [17]. Nitrogen availability and supply vary by crop species and are determined by their requirements [18]. While the world’s population continues to grow, the global demand of nitrogen (N) fertilizers is unlikely to decrease [19]. Urea and urea-containing N fertilizers are the most commonly used inorganic N fertilizers. Urea accounts for approximately 56% of global N fertilizer production [20,21]. Urea is a solid fertilizer with high N content (46%). It is easily kept and applied to crops, and it can be mixed into the soil with other N fertilizers. Nitrogen losses due to ammonia volatilization are substantial when urea-based fertilizers are used. Ammonium is transformed to ammonia and lost in the atmosphere during volatilization. Between 2006 and 2016, the annual growth in urea production was 2.8% [21].

In order to increase the efficiency of N use, in addition to good agricultural practices (for instance, proper application procedures, optimum timing, and soil testing to estimate the amount of fertilizer needed, which may be limited by physical conditions), the use of N stabilizers and nitrification inhibitors may potentially delay detrimental processes such as NH_3_ volatilization, nitrate (NO_3_^−^) leaching, and N_2_O emissions reduction [22]. A number of chemical compounds that can be added to urea to postpone the transition of N have been discovered. These slow-release products are divided into two categories: (a) urease inhibitors and (b) nitrification inhibitors. Urease inhibitors slow urea hydrolysis in soil by lowering NO_3_^–^ and NH_4_^+^ production. In addition, the presence of the inhibitor in the soil influences the effectiveness of NH_3_ loss management [22,23]. Concerning nitrification inhibitors, these have a significant impact on the enzymatic activity of NH_3_ oxidizing bacteria [24] and their addition to urea delays the conversion of ammonium ions (NH_4_^+^) to NO_3_^–^ NO_3_, potentially reducing N_2_O emissions from soil denitrification [25]. According to several researchers, urease inhibitors when added to urea reduced ammonia loss and thus increased crop seed and biomass yield and N uptake, in contrast with single urea application [26,27]. In addition, since N is a component of the chlorophyll structure, the addition of nitrification inhibitors increases the chlorophyll content in the leaves and consequently the biomass and seed yield and quality of the crops [22].

Forage constitutes an essential component of dairy ration [28]. Forage provides an effective fiber source in dairy rations, accounting for 75% of the ration’s neutral detergent fiber [29]. It should be emphasized that a lack of dietary fiber is linked to low milk fat, rumen acidity, and digestive inefficiencies [30]. Since black mustard is primarily grown for seed production, there is no information on biomass quality or forage potential. Furthermore, several research studies predict that the effect of climate change will result in large reductions in crop productivity in the Mediterranean region, and a strategy to meet the increasing demand for feed production includes the introduction of alternative crops, such as black mustard, which has beneficial properties with adequate yield and good nutritional value [31,32]. In addition, more research is needed in order to find alternative fertilization options, including slow-release inorganic nitrogen fertilizers or organic fertilizers such as compost, for the nitrogen fertilization of crops. The objective of the current study was to assess the effect of plant density and fertilization (organic with compost and inorganic using urea with and without urease and nitrification inhibitors) on aerial biomass yield and quality of the black mustard crop in order to establish alternative forage sources for livestock feed.

## 2. Results

According to the results of the two-year data analysis (Table 1), the interaction of plant density × fertilization was significant on crude ash (CA), total carbohydrate (CHO), and non-fiber carbohydrates (NFC). The main effects of plant density were significant for dry matter (DM) yield, DM content and crude protein (CP) content of black mustard aerial biomass. In response to the effect of fertilizers application, the different regimes had significant impacts on the productivity and chemical composition of biomass, excluding the crude fat (CF) and CA content (Table 1).

### 2.1. Dry Matter Yield

As shown in Table 2, the dry matter yield of black mustard was significantly affected by plant density and presented a similar trend in both years (*p* < 0.05), with the highest values presented in high-density plants (76 plants m^−2^). In the first (2019–2020) growing season, the DM yield of high-density plants was higher than that of low-density (46 plants m^−2^) by 18.49%, while during the second (2020–2021) cropping season, the DM yield of high-density was 17.26% higher than that of low-density plants. In the same manner, throughout the growing periods, the highest yields were found in plots fertilized with inorganic fertilizers. Specifically, the highest values were noticed in urea fertilizer with nitrification and urease inhibitors (U + NI + UI) (17.01 and 16.14 tn ha^−1^ for the first and second experimental year, respectively) which had no statistically significant differences with urea without inhibitors (16.23 and 16.14 tn ha^−1^ for the respective years).

### 2.2. Qualitative Characteristics of Above-Ground Biomass

The effects of plant density and fertilization on the DM content of black mustard biomass are presented in Table 2. In the low-density plants, the values of DM content were substantially higher (23.58% and 23.61% in the first and second experimental year, respectively) than those of high-density treatment (21.43% and 21.60% for the respective years). In addition, the mean values of DM content provided good evidence of the effects of different fertilization regimes. Averaged over plant densities, the highest values were presented in urea with inhibitors (27.09% and 26.03% in 2019–2020 and 2020–2021, respectively) followed by urea without inhibitors (24.94% and 25.09% in the first and second cropping season, respectively).

According to the combined analysis of variance (Table 1 and Table 2), crude protein was significantly affected by both plant density and fertilization. Concerning the plant density effect, the values of CP content in low-density plots (20.62% and 21.37% of DM in 2019–2020 and 2020–2021, respectively) were higher than in high-density plots (18.71% and 19.17% of DM in the first and second experimental year, respectively). As for the effect of fertilizers application, the highest values of CP content were observed in plots with urea with nitrification and urease inhibitors (22.80% and 23.03% of DM in 2019–2020 and 2020–2021, respectively) and urea without inhibitors (21.41% and 21.63% of DM for the respective years).

CP yield in the aerial biomass was estimated by multiplying the CP content of the aerial biomass and the aerial DM yield. According to the combined analysis of variance (Table 1), CP yield was only affected by the different fertilization treatments. During the two-year experiment, the mean values of CP yield were greatest in the urea with inhibitor treatment (3.88 and 3.70 tn ha^−1^ in the first and second experimental years, respectively) followed by urea without inhibitors (3.47 and 3.27 tn ha^−1^ in the first and second experimental years, respectively), while the lowest values (1.14 and 1.40 tn ha^−1^ for the respective years) were observed in the untreated (control) plots (Table 2).

Concerning the crude fat content, there were no significant differences among the two examined plant densities; however, the plants of low-density treatment presented slightly higher values (2.66% and 2.62% of DM in the first and second experimental years, respectively) in comparison with those of the high-density treatment (2.58% and 2.46% of DM for the respective years) (Table 3). In the same manner, the effect of fertilizer application was not statistically significant during the 2-year study, although, slightly higher values (2.68% and 2.63% of DM in 2019–2020 and 2020–2021 growing seasons, respectively) were found in the plots fertilized with the seaweed compost.

The results of the current study indicated that the crude ash content of black mustard aerial biomass was not influenced by plant density during the experimental periods (Table 3); however, the low-density plots achieved slightly higher values of this trait (14.05% and 13.95% of DM in the first and second experimental periods, respectively) than those of the high-density treatment (13.89% and 13.63% of DM for the respective experimental periods). In response to fertilization, this had also a non-significant effect on CA content, although slightly higher values (14.32% and 14.27% of DM in the first and second experimental periods, respectively) were observed in plots fertilized with urea with nitrification and urease inhibitors.

Neutral detergent fiber (NDF) content was not influenced by the different plant densities; it was only affected by the different fertilization treatments (Table 3). Specifically, the highest NDF content was observed in the case of fertilization with compost, with the values being 47.11% and 47.55% of DM in the first and the second experimental periods, respectively, while the lowest values (41.20% and 43.17% of DM for the respective experimental periods) were obtained from the untreated (control) plots.

Acid detergent fiber (ADF) did not differ among plant densities during the experimental periods (Table 3), although the plants of low-density treatment presented slightly higher values (36.34% and 37.74% of DM in the first and second growing seasons, respectively) than in high-density plots (35.28% and 36.33% of DM for the respective growing seasons). Concerning the fertilization effect, the highest ADF content values, averaged over experimental years and plant density treatments, was achieved in compost (38.98% of DM) followed by urea with inhibitors (37.21% of DM), while the lowest value (34.02% of DM) was found in the control plot.

Total carbohydrate content was only affected by fertilization during the two experimental periods (Table 4). In particular, the highest CHO content values were achieved in untreated (control: 68.25% and 67.25% of DM in the first and second growing seasons, respectively), while the lowest values (60.21% and 60.08% of DM for the respective experimental periods) were obtained in plots fertilized with urea with inhibitors.

Concerning the non-fiber carbohydrate content, there were no significant differences among the high- and low-density plots, but fertilization had a statistically significant impact on this trait. Specifically, the NFC content decreased with the increasing levels of applied nitrogen, and the highest values (control: 27.05% and 24.09% of DM in the first and second experimental year, respectively) obtained in the unfertilized (control) plots (Table 4).

## 3. Discussion

Dry matter yield of black mustard presented the highest values at the high-plant density of 76 plants m^−2^ (30 cm row spacing) with a 2-year average value being 17.98% higher as compared to the low-density of 46 plants m^−2^ (45 cm row spacing). Seed row spacing is an agronomical management strategy used by producers to optimize soil and plant ecosystem husbandry during the growth season with the aim of increasing crop production. Crop row spacing determines canopy architecture, a distinguishing feature that effects the utilization of light, water, and nutrients [33]. Narrower row spacings improve light interception, reduce intra-row competition between plants, and can improve weed management by boosting crop competitiveness and decreasing light transmittance to the soil [34,35]. The results of the current study are in line with those of Kuai et al. [36], who confirmed that narrower row spacings resulted in higher DM yield in rapeseed (*Brassica napus* L.) crop. In terms of fertilization, the highest mean values of the two experimental years were found in urea fertilizer with and without nitrification and urease inhibitors treatments (16.58 and 16.19 tn ha^−1^, respectively) presenting no statistically significant differences. According to several research studies, crops fertilized with inorganic fertilizers produced greater DM yields because these fertilizers contained soluble inorganic nitrogen that was readily available to crops, resulting in higher yields [37,38,39].

Whole-plant black mustard forage DM content decreased linearly as plant density increased, with the 2-year average values ranging from 23.60% at lower plant density (46 plants m^−2^) to 21.52% at higher plant density (76 plants m^−2^). Low planting density raises soil temperature by increasing light intensity, which raises water losses from the soil surface [33,40]. In a previous research work studying the influence of planting density on soybean physiology, there were decreased stomatal conductance and transpiration rates in high planting densities compared with lower planting densities [41]. As a result, in the current study, this phenomenon may have decreased the DM content as planting density increased, decreasing the DM content of the whole plant. Concerning the fertilization effect, the highest values of DM content were found after the application of inorganic fertilizers and specifically after the application of urea with and without inhibitors (Table 2). In particular, the highest 2-year average was found in urea with inhibitors treatment (26.56%) followed by urea without inhibitors (25.02%). The DM content presented a positive linear response to increasing available nitrogen. This response occurred because nitrogen stimulates metabolism and plant maturity, resulting in the increased accumulation of photoassimilates and their transformation into plant organs [38,39].

In order to limit nitrogen loss in the soil, higher-efficiency fertilizers coated with urease or nitrification inhibitors might be utilized. Several research studies suggested that using urease or nitrification inhibitors in conjunction with nitrogen fertilizers is one of the most promising new strategies to prevent nitrogen loss [22,42]. Using inhibitors in conjunction with nitrogen fertilizers has proven to be an extremely effective technique for decreasing nitrogen fertilizer losses and enhancing plant development and productivity [43]. In a recent study, the use of inhibitors increased maize DM content by 5–10% [44].

Biomass protein content varies with plant species, soil fertility, and crop maturity stage. In particular, as a crop reaches its full maturity, the crude protein content of the biomass as well as its digestibility as forage decreases, in contrast to the crude fiber content, which increases [45]. The CP content of alfalfa biomass ranges from 18 to 25% of DM [46]. In the current study, the results presented that the CP content of black mustard biomass was significantly influenced by both plant density and fertilization, and the maximum CP content was achieved at the early flowering stage, specifically at 120 days after sowing (DAS). Indeed, several studies have found that plant maturity can affect the protein content of forages [47,48]. According to Throop [49], the protein content of wheat biomass gradually decreased with the delay of harvest time due to the inhibition of protein synthesis by the weak photosynthetic capacity at more mature stages.

Regarding the effect of plant density, the 2-year average value of CP content recorded in the plants of the low-plant density (20.99% of DM) was higher than that in the high-density plants (18.94% of DM). The decrease in biomass protein content at high seeding density may be due to the fact that the high competition created for available resources (water and nutrients) made individual plants weaker. Additionally, high competition combined with high plant density makes less energy available for converting nitrogen into protein [50]. Increasing levels of available nutrients generally increases feed quality parameters such as CP content. Regarding the effect of fertilization, the highest 2-year average was found in urea with nitrification and urease inhibitors (22.92% of DM) followed by urea without inhibitors (21.52% of DM). The higher protein content after the application of inorganics fertilizers may be due to the fact that the higher levels of nitrogen available to plants enhance nitrogen uptake, which plays a pivotal role in protein synthesis [51]. In addition, the use of urease inhibitors reduces urea hydrolysis, adjusting the release of nitrogen mineral forms to match crop demand and therefore increasing the nitrogen use efficiency (NUE) of the crop [52].

The yield of total aboveground biomass in protein (biomass protein yield) is a function of the biomass DM yield and its protein content. As a consequence, DM yield and CP content had a strong positive correlation with the total biomass CP yield (*r* = 0.9546, *p* < 0.001 and *r* = 0.7565, *p* < 0.001, respectively; Table 5).

Crude fat is composed of lipids (galactolipids, triglycerides, and phospholipids) as well as any other non-polar compounds such as phosphatides, steroids, pigments, fat-soluble vitamins, and waxes. The percentage of CF in feed crops is typically low, with values less than 3% of DM [53]. In the current study, the CF content of black mustard biomass was not significantly affected by fertilization during the experimental periods (Table 2); however, slightly lower values were found in urea treatment without inhibitors compared with compost and urea fertilization with double inhibitors. This response could be attributed to the fact that nitrogen fertilizer increases the amount of pigments in the plant, and as conventional urea has higher volatilization, this increase in pigmentation must have been impaired, reducing the CF content in the plant [54,55,56].

The total mineral content of feed comprises crude ash, which includes inorganic compounds derived from plant and soil contaminants. High CA content indicates significant soil contamination, which can significantly increase the amount of insoluble fiber substances in NDF solution. The average ash content of alfalfa is about 11% of DM and 9% of DM for grass fodder [46]. In the present study, the combined analysis of variance showed that this trait was not influenced by the evaluated factors (Table 1); however, concerning the fertilization effect, a slightly higher 2-year value (14.30% of DM) was observed in plots fertilized with urea with nitrification and urease inhibitors. A similar trend, presenting higher values of CA content under inorganic fertilization, was also observed in quinoa (*Chenopodium quinoa* Willd.) [57] and nigella (*Nigella sativa* L.) crop [58]. In a previous study, increasing levels of inorganic fertilizer up to 150% of the recommended dose (120 kg N + 60 kg P_2_O_5_ ha^−1^) in maize crop resulted in a significant enhancement in ash content (up to 8.6% of DM) due to the high availability of the main nutrients that promote plant growth and increase the biomass DM [59]. On the contrary, fertilizer application resulted in decreased CA content in giant reed (*Arundo donax* L.) compared with unfertilized crop [60].

The content of fibrous substances or total cellulose (crude fiber) is considered a key indicator of the chemical composition when determining the nutritional value of animal feed. A high content of fibrous substances is an indication of low digestibility and energy value of the feed [61]. Crude fiber can be divided into two categories, neutral detergent fiber and acid detergent fiber. The determination of NDF and ADF is promoted with the aim of predicting DM intake and estimating digestible energy content, as well as ensuring adequate fiber in the diet of farm animals [62]. The NDF and ADF content of feed is critical, especially for lactating ruminants, because milk fat content is proportional to their percentages. For dairy cows, the US National Research Council (NRC) recommends a minimum dietary NDF content of 25 to 28% of DM and an ADF content of 17 to 21% of DM [63].

NDF approximates total cellulose by estimating cell wall components (hemicellulose, cellulose, and lignin) [64]. Rumen microorganisms breakdown hemicellulose and cellulose slowly, while lignin is indigestible. Lignin also attaches to other components of the cell wall, rendering them indigestible as well. Depending on the plant species and level of maturity, NDF is partially digested. This measure rises as plants mature and is used to predict feed intake [46,58,65]. NDF was only affected by fertilization in this study, and the highest value was reported in compost followed by urea with inhibitors treatment. The large changes in biomass NDF concentration between fertilization treatments can be attributed to fertilization’s increasing impacts on biomass fiber content [66]. So far, nitrogen fertilization treatment has been reported to have no effect on the NDF content of plant biomass [67] or to have a negative effect [56]. This disparity is related to the season of evaluation, specifically during vegetative growth. Higher growth rates are well known to cause stem accumulation and thus an increase in NDF concentration [68].

ADF is a measure of the plant’s cellulose and lignin content, and it is also partially digestible. Because high levels are associated with poor digestibility, ADF has been used to predict forage digestibility [65]. ADF, like NDF, was only influenced by fertilization and the greatest ADF value was discovered in the case of compost fertilization. In the same manner, Kering et al. [69] discovered that nitrogen fertilization reduced ADF concentration in wild horsetail (*Cynodon dactylon* (L.) Pers.).

As mentioned above, NDF estimates all cell wall components (hemicellulose, cellulose and lignin), while ADF determines cellulose and lignin. Therefore, a strong positive correlation between NDF and ADF was expected, as found in the present research work (*r* = 0.7171, *p* < 0.001; Table 5). Moreover, DM yield was positively related to NDF (*r* = 0.2874, *p* = 0.0341) and ADF (*r* = 0.2042, *p* = 0.0395) (Table 5), possibly because as black mustard plants grew, the leaf-to-stem ratio decreased, resulting in more structural tissue (total cellulose) produced to support plant growth. Similar results were also observed in alfalfa crop [70].

Carbohydrates are the primary energy source for rumen microorganisms and constitute the main energy reserve of plants, accounting for 50 to 80% of total plant energy reserves [71]. Carbohydrates provide energy to animals, with the majority of digestion occurring in the rumen in the case of ruminants. In our study, total CHO levels (58.70%–71.27% of DM) were within this range. Specifically, the total CHO content decreased linearly with the increasing levels in suppling nitrogen to the plant (Table 4) on account of the increased use of these carbohydrates to transform the available nitrogen into protein, as a plant natural response [72,73]. Our results are in agreeance with those of Leite et al. [74], who observed low total CHO when the highest nitrogen fertilization rate (270 kg N ha^−1^) applied on Marandu palisadegrass. 

The non-fiber carbohydrates fraction (starch, sugars, pectins, and β-glucans) degrades quickly in the rumen and is required to maintain adequate carbohydrate and protein degradation synchrony as well as to promote adequate microbial growth [71]. The results of the NFC content of black mustard aerial biomass are presented in Table 4. During the experimental periods, the NFC content exhibited a similar behavior as total CHO content, presenting decreases in its values with the increasing levels of available nitrogen to the plant [74]. This is also supported and confirmed by the significant and high linear correlation between the CHO and NFC contents (*r* = 0.8172, *p* < 0.001; Table 5).

## 4. Materials and Methods

### 4.1. Site Description and Experimental Design

**Figure 1:**
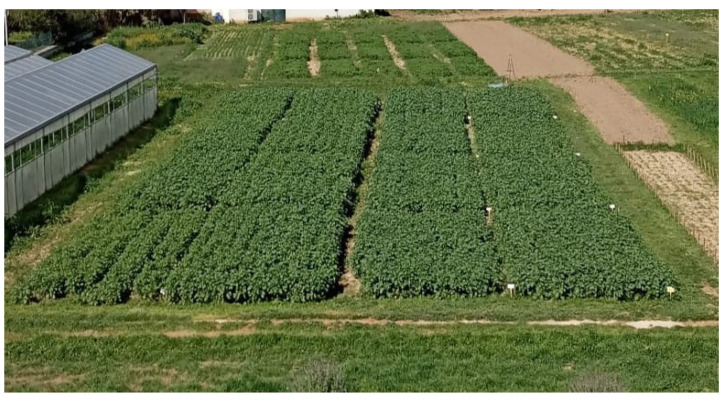
Overview of the black mustard experimental field taken from the southeast on 24 March 2021 (120 days after sowing).

**Figure 2:**
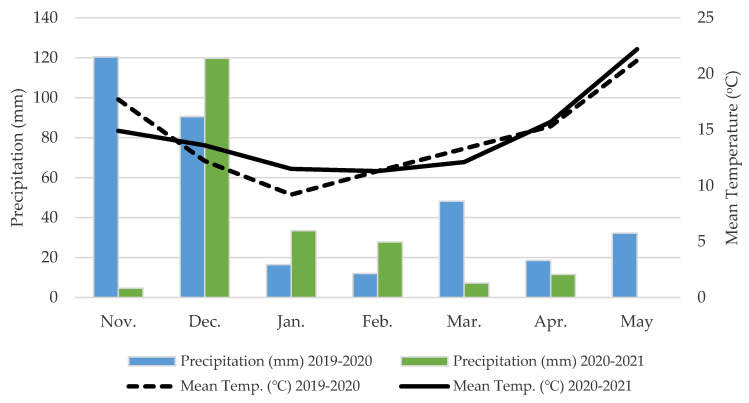
Meteorological data (mean monthly air temperature and precipitation) for experimental site throughout the duration of the 2-year study (November-May 2019–2020 and 2020–2021).

A 2-year field trial was established in the experimental field of the Agricultural University of Athens (AUA) (37°59′ N and 23°42′ E; 30 m altitude) during the cropping periods 2019–2020 and 2020–2021 (Figure 1). The main soil properties (at 0–30 cm sampling depth) of the experimental site are demonstrated as follows: the soil was a clay loam (29.4% clay, 35.1% silt, and 35.5% sand) with a pH of 7.39 (1:1 H_2_O), total nitrogen (N) percentage of 0.143%, available phosphorus (Olsen P) of 13.6 mg kg^−1^ soil, available potassium (K) of 233 mg kg^−1^ soil, calcium carbonate (CaCO_3_) percentage of 15.34%, and soil organic matter percentage of 1.67%. The weather data were measured throughout the experimental periods, including mean monthly air temperature and rainfall being obtained from an automatic weather station (Davis Vantage Pro2 Weather Station; Davis Instruments Corporation, Hayward, CA, USA), located at the experimental field of AUA, and are presented in Figure 2. Total rainfall in the 2019–2020 and 2020–2021 cropping periods (from November to May) was 338.4 and 204.4 mm, respectively. The mean air temperature during the growing periods was 14.3 °C for 2019–2020 and 14.4 °C for 2020–2021.

The experiment was set up on a 1015 m^2^ area and arranged in a split-plot design, with 3 replications, in a 2 × 4 factorial scheme. The whole-plot factor was two different plant densities (PD1: 46 plants m^−2^ and PD2: 76 plants m^−2^), and the split-plot factor was four different fertilization types (untreated (Control), urea (U), urea with Nitrification and Urease Inhibitors (U + NI + UI) and seaweed compost (Compost)). The rate of each type of fertilizer used in the current study is the general recommended dose of the corresponding type of fertilizer for black mustard production in clay–loam soils [8,10,32]. Specifically, the total applied fertilizer dose for urea fertilizers with and without inhibitors was 140 kg N ha^−1^, while the nitrogen application rate for seaweed compost was 50 kg N ha^−1^. The type of urea fertilizer was 46-0-0. The nitrification inhibitor was N-((3(5)-methyl-1H-pyrazol-1-yl) methyl) acetamide (MPA; 0.07%) and the urease inhibitor was N-(2-Nitrophenyl) phosphoric triamide (2-NPT; 0.035%) for the fertilizer with urea with double inhibitors (46-0-0). As for the seaweed compost, the N content was 1.98%. The main plot and sub-plot sizes were 140 m^2^ (35 m × 4 m) and 32 m^2^ (8 m × 4 m), respectively. During each cultivation period, three days prior to the sowing, the soil was prepared by mouldboard ploughing at a depth of 25 cm. Fertilizers were applied as basal dressing through broadcasting by hand and incorporated with the soil by harrowing. Black mustard [*Brassica nigra* (L.) Koch] seeds were broadcasted by hand in rows 45 and 30 cm apart for PD1 and PD2, respectively, and 15 cm within each row. Seed sowing was performed on 29 and 24 November for the first and the second experimental year, respectively. The crop was harvested by hand on 6 June 2020 and 24 May 2021, when the seeds reached full maturity (seed moisture was approximately 9%). During the cropping periods, there was no incidence of pests or disease in the black mustard crop. Moreover, weeds were controlled by hand-hoeing when needed and before canopy closure.

### 4.2. Sampling Procedures, Measurements and Methods

A total of 20 plant samples were randomly collected from each sub-plot at the early flowering stage of black mustard, specifically at 120 days after sowing (DAS). Above-ground dry matter (DM) was determined after drying for 48 h at 64 °C. Then, the DM weight of the whole plant and the yield per unit area were measured and calculated according to the planting density.

As for the chemical composition analysis, the plant samples were ground to pass through a 1-mm Wiley mill (Thomas T4274.E15 Steel Model 4 Wiley Mill; Arthur H. Thomas, Philadelphia, PA, USA) screen for chemical analysis. The samples were analyzed for DM analysis (method 943.01), crude ash (CA; method 924.05), Kjeldahl nitrogen (CP; method 984.13) using a Kjeltec 8400 auto-analyzer (Foss Tecator AB, Höganas, Sweden), crude fat (CF; method 920.39) and acid detergent fiber (ADF; method 973.18) according to the Association of Official Analytical Chemists [75]. The crude protein (CP) content was calculated by multiplying the Kjeldahl nitrogen concentration by 6.25. Neutral detergent fiber (NDF; method 930.15) was determined using heat stable amylase according to the procedure of Van Soest et al. [28]. An ANKOM 200 Fiber Analyzer (ANKOM Technology Corporation, NY, USA) was used for the determination of ADF and NDF. In addition, the total carbohydrate (CHO) and non-fiber CHO (NFC) were calculated as follow according to National Research Council [63]:

Total carbohydrate (CHO) = 100 − (CF + CP + CA) (%)(1)

Non-Fiber CHO (NFC) = 100 − (CP + NDF + CF + CA) (%)(2)

### 4.3. Statistical Analysis

Statistical analysis was performed using the SigmaPlot 12 statistical software (Systat Software Inc., San Jose, CA, USA). The analysis of variance (ANOVA) used a mixed model, with years and replications as random effects and plant density and fertilization as fixed effects. The estimation of significant differences among the treatments were determined using Tukey’s honestly significant difference test (Tukey’s HSD). Correlation analyses were used to describe the relationships between the yield components and nutritive characteristics using Pearson’s correlation. For all statistical analyses in the current study, significance was declared at 5% (*p* ≤ 0.05).

## 5. Conclusions

The results indicated that black mustard aerial DM yield was influenced by both plant density and fertilization. The highest DM yield was observed in high-density (76 plants m^−2^) plots fertilized with urea fertilizer coated with double (nitrification and urease) inhibitors. In terms of the qualitive parameters of total above-ground biomass, the highest CP content was achieved in plots with low density (46 plants m^−2^) and inorganic fertilization with urea with double inhibitors. Moreover, NDF and ADF were not affected by plant density; however, there were significant differences between fertilization treatments. The highest ADF and NDF contents of above-ground biomass were found under compost and urea with double inhibitors. The high ADF, NDF, and relatively high CP content characterized black mustard aerial biomass can meet the requirements of lactating animals, and therefore the production of black mustard biomass as forage crop could be of great importance. As a conclusion, black mustard cultivated at plant densities higher than 46 plants m^−2^ and under inorganic fertilization, especially with urea coated with double inhibitors, could be successfully used as a novel forage crop in ruminants’ diets. In addition, future studies should be directed toward understanding the effect of the use of higher-efficiency fertilizers coated with urease and/or nitrification inhibitors on the quality of alternative forage crops, as black mustard, and their consumption on the growth performance and health of ruminants.

## Figures and Tables

**Table 1:** Combined analysis of variance (*F*) for all measured traits of black mustard biomass in two experimental years.

Source of Variance	Df	DM Yield	DM Content	CP Content	CP Yield	CF
Year (Y)	1	0.0248 ^ns^	0.0040 ^ns^	0.9768 ^ns^	0.0524 ^ns^	1.6580 ^ns^
Plant Density (PD)	1	9.9495 **	10.2966 **	11.1732 **	2.5311 ^ns^	3.4333 ^ns^
Fertilization (F)	3	37.3676 ***	37.5041 ***	22.9083 ***	58.1784 ***	0.7898 ^ns^
Y × PD	1	0.0134 ^ns^	0.0110 ^ns^	0.0575 ^ns^	0.0859 ^ns^	0.3267 ^ns^
Y × F	3	0.4005 ^ns^	0.6889 ^ns^	0.1593 ^ns^	0.7508 ^ns^	0.1106 ^ns^
PD × F	3	0.5494 ^ns^	0.4754 ^ns^	4.9791 ^ns^	2.3535 ^ns^	2.2104 ^ns^
Y × PD × F	3	0.0318 ^ns^	0.0176 ^ns^	0.0769 ^ns^	0.0072 ^ns^	0.1207 ^ns^
**Source of Variance**	**Df**	**CA**	**NDF**	**ADF**	**CHO**	**NFC**
Year (Y)	1	0.3525 ^ns^	0.8023 ^ns^	3.5077 ^ns^	0.2103 ^ns^	1.4313 ^ns^
Plant Density (PD)	1	0.6374 ^ns^	3.8650 ^ns^	3.5698 ^ns^	3.6233 ^ns^	2.1571 ^ns^
Fertilization (F)	3	1.1141 ^ns^	7.2726 ***	9.4025 ***	11.8053 ***	19.0120 ***
Y × PD	1	0.0723 ^ns^	0.2196 ^ns^	0.0711 ^ns^	0.1302 ^ns^	0.0173 ^ns^
Y × F	3	0.0477 ^ns^	0.3641 ^ns^	0.5735 ^ns^	0.0901 ^ns^	0.5744 ^ns^
PD × F	3	2.9494 *	1.6596 ^ns^	1.5429 ^ns^	3.2970 *	4.5739 *
Y × PD × F	3	0.0214 ^ns^	0.1033 ^ns^	0.0650 ^ns^	0.0452 ^ns^	0.1005 ^ns^

*F*-test ratios are from ANOVA. ns, *, ** and ***: Not significant and significant at 5%, 1%, and 0.1% probability levels, respectively. Df: Degrees of freedom; DM: Dry matter; CP: Crude protein; CF: Crude fat; CA: Crude ash; NDF: Neutral detergent fiber; ADF: Acid detergent fiber; CHO: Total carbohydrate; NFC: Non-fiber CHO.

**Table 2:** Dry matter (DM) yield, DM content, crude protein (CP) content, and CP yield as influenced by the plant density and fertilization.

Plant Density (Plants m^−2^)												
**Fertilization**	46	76		46	76		46	76		46	76	
	**DM Yield**			**DM Content (%)**			**CP Content**			**CP Yield**		
**2019–2020**	**(tn ha^−1^)**		*Mean*			*Mean*	**(% of DM)**		*Mean*	**(tn ha^−1^)**		*Mean*
Control	7.08	7.82	7.45 c	17.57	16.85	17.21 c	18.15	12.98	15.57 c	1.29	0.99	1.14 c
Urea	14.99	17.48	16.23 a	25.89	23.99	24.94 a	22.03	20.79	21.41 a	3.31	3.63	3.47 a
Urea + NI + UI	15.68	18.34	17.01 a	28.43	25.76	27.09 a	22.09	23.51	22.80 a	3.45	4.31	3.88 a
Compost	9.42	12.24	10.83 b	22.45	19.12	20.79 b	20.21	17.58	18.89 b	1.90	2.15	2.02 b
*Mean*	11.79 B	13.97 A		23.58 A	21.43 B		20.62 A	18.71 B		2.49 A	2.77 A	
*F_Plant Density_*	5.2173 * (Tukey = 1.707)			4.7546 * (Tukey = 1.664)			5.6664 * (Tukey = 1.101)			1.7033 ^ns^		
*F_Fertilization_*	22.7083 *** (Tukey = 2.176)			19.8362 *** (Tukey = 3.035)			15.7790 *** (Tukey = 1.529)			34.1834 *** (Tukey = 0.662)		
*F_Plant Density_* × *_Fertilization_*	0.2566 ^ns^			0.3231 ^ns^			2.9523 ^ns^			1.1902 ^ns^		
**2020–2021**												
Control	7.99	8.84	8.42 c	18.51	17.69	18.10 c	19.82	13.81	16.82 c	1.60	1.21	1.40 c
Urea	14.18	16.09	15.14 a	26.05	24.14	25.09 a	22.20	21.07	21.63 ab	3.15	3.38	3.27 a
Urea + NI + UI	14.73	17.55	16.14 a	27.43	24.63	26.03 a	22.77	23.29	23.03 a	3.35	4.06	3.70 a
Compost	10.36	12.95	11.65 b	22.45	19.98	21.21 b	20.71	18.53	19.62 b	2.17	2.40	2.29 b
*Mean*	11.82 B	13.86 A		23.61 A	21.60 B		21.37 A	19.17 B		2.57 A	2.76 A	
*F_Plant Density_*	5.0832 * (Tukey = 1.110)			5.2728 * (Tukey = 1.216)			5.5777 * (Tukey = 0.648)			0.8791 ^ns^		
*F_Fertilization_*	15.0869 *** (Tukey = 2.788)			17.661 *** (Tukey = 1.759)			8.3990 ** (Tukey = 2.321)			24.3321 *** (Tukey = 0.621)		
*F_Plant Density_* × *_Fertilization_*	0.2353 ^ns^			0.2488 ^ns^			2.2147 ^ns^			1.1696 ^ns^		

*F*-test ratios are from ANOVA. ns, *, ** and ***: Not-significant and significant at 5%, 1% and 0.1% probability levels, respectively. The capital letters compare plant densities within a growing season, and lowercase letters compare fertilization treatments within a growing season, by Tukey’s HSD test (*p* ≤ 0.05).

**Table 3:** Crude fat (CF), crude ash (CA), neutral detergent fiber (NDF), and acid detergent fiber (ADF) content as influenced by the plant density and fertilization.

Plant Density (Plants m^−2^)												
**Fertilization**	46	76		46	76		46	76		46	76	
	**CF (% of DM)**			**CA (% of DM)**			**NDF (% of DM)**			**ADF (% of DM)**		
**2019–2020**			*Mean*			*Mean*			*Mean*			*Mean*
Control	2.64	2.53	2.59 a	13.99	13.22	13.61 a	43.51	38.88	41.20 c	33.37	31.94	32.66 c
Urea	2.67	2.53	2.60 a	14.48	13.57	14.03 a	43.71	43.79	43.75 bc	36.20	34.57	35.39 bc
Urea + NI + UI	2.76	2.50	2.63 a	13.61	15.03	14.32 a	47.63	44.53	46.08 ab	38.02	35.66	36.84 ab
Compost	2.59	2.76	2.68 a	14.12	13.73	13.93 a	47.28	46.94	47.11 a	37.77	38.92	38.35 a
*Mean*	2.66 A	2.58 A		14.05 A	13.89 A		45.53 A	43.54 A		36.34 A	35.28 A	
*F_Plant Density_*	0.7386 ^ns^			0.1178 ^ns^			3.7800 ^ns^			1.2396 ^ns^		
*F_Fertilization_*	0.1596 ^ns^			0.3880 ^ns^			6.5442 ** (Tukey = 2.632)			6.4133 ** (Tukey = 2.762)		
*F_Plant Density_* × *_Fertilization_*	0.8459 ^ns^			1.3120 ^ns^			1.1939 ^ns^			0.6393 ^ns^		
**2020–2021**												
Control	2.58	2.35	2.46 a	13.88	13.05	13.47 a	44.54	41.78	43.17 b	36.77	33.99	35.38 b
Urea	2.63	2.31	2.47 a	14.31	13.41	13.86 a	44.72	44.66	44.69 ab	36.92	35.27	36.10 b
Urea + NI + UI	2.71	2.45	2.58 a	13.66	14.87	14.27 a	47.29	44.05	45.67 ab	38.77	36.37	37.57 ab
Compost	2.54	2.71	2.63 a	13.95	13.17	13.56 a	46.98	48.12	47.55 a	38.51	39.69	39.10 a
*Mean*	2.62 A	2.46 A		13.95 A	13.63 A		45.89 A	44.66 A		37.74 A	36.33 A	
*F_Plant Density_*	3.3081 ^ns^			0.7034 ^ns^			1.9219 ^ns^			2.4782 ^ns^		
*F_Fertilization_*	0.8138 ^ns^			0.8646 ^ns^			4.0608 * (Tukey = 2.979)			3.3738 * (Tukey = 2.291)		
*F_Plant Density_* × *_Fertilization_*	1.5655 ^ns^			1.7403 ^ns^			0.6901 ^ns^			0.9904 ^ns^		

*F*-test ratios are from ANOVA. ns, *, **: Not significant and significant at 5%, 1% probability levels. The capital letters compare plant densities within a growing season, and lowercase letters compare fertilization treatments within a growing season, by Tukey’s HSD test (*p* ≤ 0.05).

**Table 4:** Total carbohydrate (CHO) and non-fiber carbohydrate (NFC) content as influenced by the plant density and fertilization.

		Plant Density (Plants m^−2^)				
**Fertilization**	46	76		46	76	
	**CHO (% of DM)**			**NFC (% of DM)**		
**2019–2020**			*Mean*			*Mean*
Control	65.22	71.27	68.25 a	21.71	24.39	23.05 a
Urea	60.73	63.14	61.94 bc	17.02	19.35	18.18 b
Urea + NI + UI	61.72	58.70	60.21 c	14.08	14.18	14.13 c
Compost	62.99	66.16	64.58 b	15.72	19.22	17.47 b
*Mean*	62.67 A	64.82 A		17.13 A	19.28 A	
*F_Plant Density_*	1.9679 ^ns^			1.6417 ^ns^		
*F_Fertilization_*	7.1572 *** (Tukey = 3.627)			10.5627 *** (Tukey = 2.303)		
*F_Plant Density_* × *_Fertilization_*	2.8491 ^ns^			3.5275 ^ns^		
**2020–2021**						
Control	63.72	70.79	67.25 a	19.17	21.01	20.09 a
Urea	60.79	63.07	61.93 bc	16.06	18.41	17.24 b
Urea + NI + UI	61.03	59.13	60.08 c	13.74	15.08	14.41 c
Compost	62.71	65.99	64.35 ab	15.73	17.87	16.80 b
*Mean*	62.06 A	64.75 A		16.18 A	18.09 A	
*F_Plant Density_*	5.6762 ^ns^			4.7703 ^ns^		
*F_Fertilization_*	7.5967 ** (Tukey = 4.050)			9.9054 *** (Tukey = 1.897)		
*F_Plant Density_* × *_Fertilization_*	2.6744 ^ns^			2.2524 ^ns^		

*F*-test ratios are from ANOVA. ns, ** and ***: Not-significant and significant at 1% and 0.1% probability levels, respectively. The capital letters compare plant densities within a growing season, and lowercase letters compare fertilization treatments within a growing season, by Tukey’s HSD test (*p* ≤ 0.05).

**Table 5:** Heatmap of correlation matrix between evaluated traits.

Trait	Coefficient of Correlaiton (*r*)										
	DM Content	DM Yield	CP Content	CP Yield	CF	CA	NDF	ADF	CHO	NFC	
DM Content	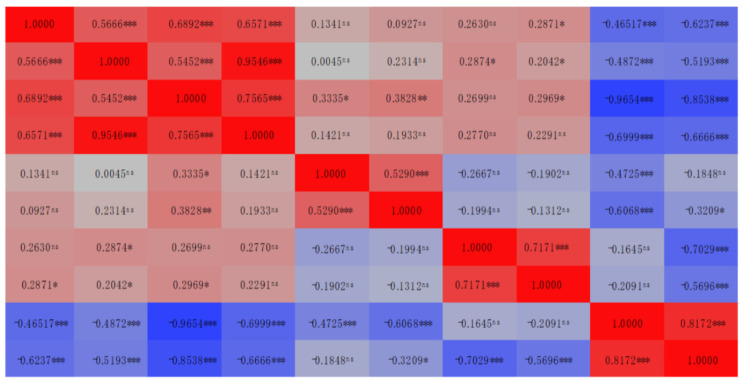										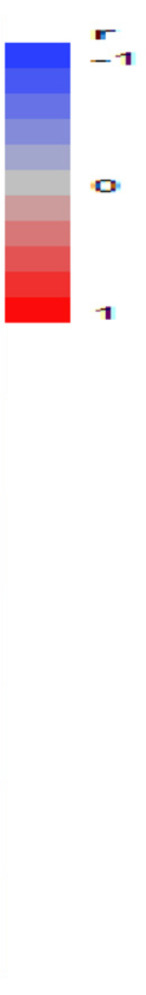
DM Yield											
CP Content											
CP Yield											
CF											
CA											
NDF											
ADF											
CHO											
NFC											

ns, *, ** and ***: Not significant and significant at 5%, 1% and 0.1% probability levels, respectively. DM: Dry matter; CP: Crude protein; CF: Crude fat; CA: Crude ash; NDF: Neutral detergent fiber; ADF: Acid detergent fiber; CHO: Total carbohydrate; NFC: Non-fiber CHO.

## Data Availability

Not applicable.

## References

[1] United Nations (UN) World Population Projected to Reach 9.8 billion in 2050, and 11.2 billion in 2100. United Nations Department of Economic and Social Affairs. https://www.un.org/en/desa/world-population-projected-reach-98-billion-2050-and-112-billion-2100.

[2] Food and Agriculture Organization of the United Nations (FAO) (2011). World Livestock 2011—Livestock in Food Security.

[3] Ponnampalam E.N., Holman B.W.B., Kerry J.P., Przybylski W., Hopkins D. (2016). Impact of animal nutrition on muscle composition and meat quality. Meat Quality: Genetic and Environmental Factors.

[4] Demeyer D., Doreau M. (1999). Targets and procedures for altering ruminant meat and milk lipids. Proc. Nutr. Soc..

[5] Kaasschieter G.A., de Jong R., Schiere J.B., Zwart D. (1992). Towards a sustainable livestock production in developing countries and the importance of animal health strategy therein. Vet. Q..

[6] Wanapat M., Kang S., Polyorach S. (2013). Development of feeding systems and strategies of supplementation to enhance rumen fermentation and ruminant production in the tropics. J. Anim. Sci. Biotechnol..

[7] Tona G.O., Yücel B., Taşkin T. (2018). Current and Future Improvements in Livestock Nutrition and Feed Resources. Animal Husbandry and Nutrition.

[8] Thomas J., Kuruvilla M.K., Hrideek K.T., Peter K.V. (2012). Mustard. Handbook of Herbs and Spices.

[9] Bagchi G.D., Srivastava N.G., Caballero B., Trugo L., Finglas P.M. (2003). Spices and flavoring crops: Fruits and seeds. Encyclopedia of Food Sciences and Nutrition.

[10] Kakabouki I., Karydogianni S., Roussis I., Bilalis D. (2020). Effect of organic and inorganic fertilization on weed flora and seed yield in black mustard (*Brassica nigra* (L.) Koch) crops. Int. J. Agric. Nat. Resour..

[11] Sahay S., Inam A., Inam A., Iqbal S. (2015). Modulation in growth, photosynthesis and yield attributes of black mustard (*B. nigra* cv. IC247) by interactive effect of wastewater and fly ash under different NPK levels. Cogent Food Agric..

[12] Angelova V., Ivanova K. (2009). Bioaccumulation and distribution of heavy metals in black mustard (*Brassica nigra* Koch). Environ. Monit. Assess..

[13] Rahman M., Khatun A., Liu L., Barkla B.J. (2018). Brassicaceae mustards: Traditional and agronomic uses in Australia and New Zealand. Molecules.

[14] Wanasundara J.P. (2011). Proteins of Brassicaceae oilseeds and their potential as a plant protein source. Food Sci. Nutr..

[15] Čolović D., Banjac V., Rakita S., Čolović R., Marjanović-Jeromela A., Vidosavljević S., Kokić B. (2018). By-products of black (*Brassica nigra*) and white (*Sinapis slba*) mustard seed production as animal feed: Possibilities and hazards. J. Process. Energy Agric..

[16] Montemurro F., Diacono M. (2016). Towards a better understanding of agronomic efficiency of nitrogen: Assessment and improvement strategies. Agronomy.

[17] Delogu G., Cattivelli L., Pecchioni N., De Falcis D., Maggiore T., Stanca A.M. (1998). Uptake and agronomic efficiency of nitrogen in winter barley and winter wheat. Eur. J. Agron..

[18] Sinclair T.R., de Wit C.T. (1975). Photosynthate and nitrogen requirements for seed production by various crops. Science.

[19] Bakken L.R., Frostegard A. (2017). Sources and sinks for N_2_O, can microbiologist help to mitigate N_2_O emissions?. Environ. Microbiol..

[20] Bremner J.M. (2007). Problems in the use of urea as a nitrogen fertilizer. Soil Use Manag..

[21] Suter H.C., Sultana H., Davies R., Walker C., Chen D. (2016). Influence of enhanced efficiency fertilisation techniques on nitrous oxide emissions and productivity response from urea in a temperate Australian ryegrass pasture. Soil Res..

[22] Wang H., Köbke S., Dittert K. (2020). Use of urease and nitrification inhibitors to reduce gaseous nitrogen emissions from fertilizers containing ammonium nitrate and urea. Glob. Ecol. Conserv..

[23] Li Y., Mingfang H., Tenuta M., Ma Z., Gui D., Li X., Zeng F., Gao X. (2020). Agronomic evaluation of polymercoated urea and urease and nitrification inhibitors for cotton production under drip-fertigation in a dry climate. Sci. Rep..

[24] Ruser R., Schulz R. (2015). The effect of nitrification inhibitors on the nitrous oxide (N_2_O) release from agricultural soils: A review. J. Plant Nutr. Soil Sci..

[25] Franzen D.W. (2022). Nitrogen Extenders and Additives for Field Crops (SF1581).

[26] Karydogianni S., Darawsheh M.K., Kakabouki I., Zisi C., Folina A.E., Roussis I., Tselia Z., Bilalis D. (2020). Effect of nitrogen fertilizations, with and without Inhibitors, on cotton growth and fiber quality. Agron. Res..

[27] Krol D.J., Forrestal J.P., Wall D., Lanigan J.G., Sanz-Gomez J., Richards G.K. (2020). Nitrogen fertilizers with urease inhibitors reduce nitrous oxide and ammonia losses, while retaining yield in temperate grassland. Sci. Total Environ..

[28] Van Soest P.J., Robertson J.B., Lewis B.A. (1991). Methods for dietary fiber, neutral detergent fiber, and non-starch polysaccharides in relation to animal nutrition. J. Dairy Sci..

[29] Mertens D.R. (1997). Creating a system for meeting the fiber requirements of dairy cow. J. Dairy Sci..

[30] Orskov E.R., Ryle M. (1990). Energy Nutrition in Ruminants.

[31] Bilalis D.J., Roussis I., Cheimona N., Kakabouki I., Travlos I.S., Gorawala P., Mandhatri S. (2018). Organic Agriculture and Innovative Feed Crops. Agricultural Research Updates.

[32] Kakabouki I., Tataridas A., Mavroeidis A., Kousta A., Roussis I., Katsenios N., Efthimiadou A., Papastylianou P. (2021). Introduction of alternative crops in the Mediterranean to satisfy EU Green Deal goals. A review. Agron. Sustain. Dev..

[33] Sharratt B.S., McWilliams D.A. (2005). Microclimatic and rooting characteristics of narrow-row versus conventional-row corn. Agron. J..

[34] Edwards J.T., Purcell L.C., Vories E.D. (2005). Light interception and yield potential of short-season maize (*Zea mays* L.) hybrids in the Midsouth. Agron. J..

[35] De Bruin J.L., Pedersen P. (2008). Effect of row spacing and seeding rate on soybean yield. Agron. J..

[36] Kuai J., Sun Y., Zuo Q., Huang H., Liao Q., Wu C., Lu J., Wu J., Zhou G. (2015). The yield of mechanically harvested rapeseed (*Brassica napus* L.) can be increased by optimum plant density and row spacing. Sci. Rep..

[37] Smith R., Slater F.M. (2010). The effects of organic and inorganic fertilizer applications to *Miscanthus* × *giganteus*, *Arundo donax* and *Phalaris arundinacea*, when grown as energy crops in Wales, UK. Glob. Chang. Biol. Bioenergy.

[38] Baghdadi A., Halim R.A., Ghasemzadeh A., Ramlan M.F., Sakimin S.Z. (2018). Impact of organic and inorganic fertilizers on the yield and quality of silage corn intercropped with soybean. PeerJ.

[39] Sandrakirana R., Arifin Z. (2021). Effect of organic and chemical fertilizers on the growth and production of soybean (*Glycine max*) in dry land. Rev. Fac. Nac. Agron. Medellín..

[40] Stickler F.C., Laude H.H. (1960). Effect of row spacing and plant population on performance of corn, grain sorghum and forage sorghum. Agron. J..

[41] Moreira A., Moraes L.A.C., Schroth G., Mandarino J.M.G. (2015). Effect of nitrogen, row spacing, and plant density on yield, yield components, and plant physiology in soybean-wheat intercropping. Agron. J..

[42] Zaman M., Nguyen M.L., Blennerhassett J.D., Quin B.F. (2008). Reducing NH_3_, N_2_O and NO_3_^−^–N losses from a pasture soil with urease or nitrification inhibitors and elemental S-amended nitrogenous fertilizers. Biol. Fertil. Soils..

[43] Drury C.F., Yang X., Reynolds W.D., Calder W., Oloya T.O., Woodley A. (2017). Combining urease and nitrification inhibitors with incorporation reduces ammonia and nitrous oxide emissions and increases corn yields. J. Environ. Qual..

[44] Drulis P., Kriauciuniene Z., Liakas V. (2022). The effect of combining N-fertilization with urease inhibitors and biological preparations on maize biological productivity. Agronomy.

[45] Rayburn E.B., Sharpe P. (2019). Matching plant species to your environment, weather, and climate. Horse Pasture Management.

[46] Horrocks R.D., Valentine J.F. (1999). Harvested Forages.

[47] Xie Z.L., Zhang T.F., Chen X.Z., Li G.D., Zhang J.G. (2012). Effects of maturity stages on the nutritive composition and silage quality of whole crop wheat. Asian-Australas. J. Anim. Sci..

[48] Krawutschke M., Kleen J., Weiher N., Loges R., Taube F., Gierus M. (2013). Changes in crude protein fractions of forage legumes during the spring growth and summer regrowth period. J. Agric. Sci..

[49] Throop H.L. (2005). Nitrogen deposition and herbivory affect biomass production and allocation in an annual plant. OIKOS.

[50] Widdicombe W.D., Thelen K.D. (2002). Row width and plant density effect on corn forage hybrids. Agron. J..

[51] Quemada M., Gabriel J.L. (2016). Approaches for increasing nitrogen and water use efficiency simultaneously. Glob. Food Sec..

[52] Allende-Montalbán R., Martín-Lammerding D., Delgado M.d.M., Porcel M.A., Gabriel J.L. (2021). Urease inhibitors effects on the nitrogen use efficiency in a maize–wheat rotation with or without water deficit. Agriculture.

[53] Coleman S.W., Henry D.A., Freer M., Dove H. (2002). Nutritive Value of Herbage. Sheep Nutrition.

[54] Dewhurst R.J., Scollan N.D., Younell S.J., Tweed J.K.S., Humphreys M.O. (2001). Influence of species, cutting date and cutting interval on the fatty acid composition of grasses. Grass Forage Sci..

[55] Boufaïed H., Chouinard P.Y., Tremblay G.F., Petit H.V., Michaud R., Bélanger G. (2003). Fatty acids in forages. I. Factors affecting concentrations. Can. J. Anim. Sci..

[56] Dasci M., Comakli B. (2011). Effects of fertilization on forage yield and quality in range sites with different topographic structure. Turkish J. Field Crop..

[57] Kakabouki I., Bilalis D., Karkanis A., Zervas G., Tsiplakou E., Hela D. (2014). Effects of fertilization and tillage system on growth and crude protein content of quinoa (*Chenopodium quinoa* Willd.): An alternative forage crop. Emir. J. Food Agric..

[58] Roussis I., Kakabouki I., Tsiplakou E., Bilalis D., Berghuis S. (2020). Influence of plant density and fertilization on yield and crude protein of *Nigella sativa* L.: An alternative forage and feed source. Nigella sativa: Properties, Uses and Effects.

[59] Reddy B.V.S., Reddy P.S., Bidinger F.R., Blümmel M. (2003). Crop management factors influencing yield and quality of crop residues. Field Crops Res..

[60] Nassi o Di Nasso N., Angelini L.G., Bonari E. (2010). Influence of fertilisation and harvest time on fuel quality of giant reed (*Arundo donax* L.) in Central Italy. Eur. J. Agron..

[61] Krachunov I. (2007). Estimation of energy feeding value of forages for ruminants II. Energy prediction through crude fiber content. J. Mt. Agric. Balk..

[62] Beauchemin K.A. (1996). Using ADF and NDF in dairy cattle diet formulation-a western Canadian perspective. Anim. Feed Sci. Technol..

[63] National Research Council (NRC) (2001). Nutrient Requirements of Dairy Cattle.

[64] Duodu K.G., Dowell F.E., Taylor J.R.N., Duodu K.G. (2019). Sorghum and Millets: Quality Management Systems. Sorghum and Millets: Chemistry, Technology and Nutritional Attributes.

[65] Han F., Ullrich S.E., Romagosa I., Clancy J.A., Froseth J.A., Wesenberg D.M. (2003). Quantitative genetic analysis of acid detergent fibre content in barley grain. J. Cereal Sci..

[66] Messman M.A., Weiss W.P., Erickson D.O. (1991). Effects of nitrogen fertilization and maturity of bromegrass on in situ ruminal digestion kinetics of fiber. J. Anim. Sci..

[67] Benett C.G.S., Buzetti S., Silva K.S., Bergamaschine A.F., Fabricio J.A. (2008). Yield and bromatologic composition of Marandu grass as function of sources and doses of nitrogen. Ciênc. Agrotec..

[68] Sbrissia A.F., da Silva S.C. (2008). Tiller size/density compensation in Marandu palisadegrass swards. Rev. Brasi. Zootec..

[69] Kering M.K., Guretzky J.A., Funderburg E., Mosali J. (2011). Effect of nitrogen fertilizer rate and harvest season on forage yield, quality, and macronutrient concentrations in Midland Bermuda grass. Commun. Soil Sci. Plant Anal..

[70] Sheaffer C.C., Martin N.P., Lamb J.F.S., Cuomo G.R., Jewett J.G., Quering S.R. (2000). Leaf and stem properties of alfalfa entries. Agron. J..

[71] Van Soest P.J. (1994). Nutritional ecology of the ruminant.

[72] Taiz L., Zeiger E. (2002). Plant Physiology.

[73] Pereira J.R., Neres M.A., Sandini I.E., Fluck A.C., Costa O.A.D., Sartor L.R. (2020). Chemical compounds and gas production kinetics of annual ryegrass hay in distinct nitrogen levels and cutting heights. Turk. J. Vet. Anim. Sci..

[74] Leite R.G., Cardoso A.D.S., Fonseca N.V.B., Silva M.L.C., Tedeschi L.O., Delevatti L.M., Ruggieri A.C., Reis R.A. (2021). Effects of nitrogen fertilization on protein and carbohydrate fractions of Marandu palisadegrass. Sci Rep..

[75] Association of Official Analytical Chemists (AOAC) (1990). Official Methods of Analysis, Association of Official Analytical Chemists.

